# Insights into the mechanisms of drug resistance in renal cell carcinoma: from multi-omics perspective

**DOI:** 10.1097/JS9.0000000000004600

**Published:** 2026-02-03

**Authors:** Jinxin Li, Jiatao Hu, Yiren Yang, Hanzhong Zhang, Ying Liu, Yu Fang, Le Qu, Anqi Lin, Peng Luo, Aimin Jiang, Linhui Wang

**Affiliations:** a Department of Urology, Changhai Hospital, Naval Medical University (Second Military Medical University), Shanghai, China; b Department of Urology, Affiliated Jinling Hospital, Medical School of Nanjing University, Nanjing, China; c Donghai County People’s Hospital - Jiangnan University Smart Healthcare Joint Laboratory, Donghai County People’s Hospital (Affiliated Kangda College of Nanjing Medical University), Lianyungang, China; d Department of Oncology, Zhujiang Hospital, Southern Medical University, Guangzhou, China

**Keywords:** drug resistance, immunotherapy, multi-omics, renal cell carcinoma, targeted therapy

## Abstract

Renal cell carcinoma (RCC) is a prevalent malignancy of the urinary system. Despite significant advances achieved through targeted therapies and immunotherapies, therapeutic resistance remains a major obstacle to sustained clinical efficacy. This review comprehensively examines the molecular mechanisms driving resistance to both targeted therapy and immunotherapy in RCC from a multi-omics perspective. By integrating findings across diverse omics layers, we underscore the pivotal role of multi-level regulatory networks in mediating drug resistance and immune evasion. Our objective is to provide an in-depth understanding of these resistance mechanisms and to establish a theoretical framework for developing innovative therapeutic strategies aimed at overcoming resistance, thereby facilitating the advancement of precision oncology in RCC.

## Introduction

Renal cell carcinoma (RCC) is one of the most prevalent malignancies of the urinary system, accounting for approximately 2%–3% of all global cancer cases. Established risk factors include smoking, obesity, and genetic predisposition^[1–4]^. According to GLOBOCAN data, more than 434 000 new RCC cases were diagnosed worldwide in 2022, resulting in nearly 156 000 deaths. Notably, RCC incidence is significantly higher in males than in females, with a male-to-female ratio of approximately 1.5:1^[1]^. While surgical resection remains the standard treatment for early-stage RCC, around 30% of patients with locally advanced disease experience postoperative recurrence. The prognosis for metastatic RCC (mRCC) remains poor, with a 5-year survival rate of less than 15%^[5,6]^.

HIGHLIGHTS

- Multi-omics approaches reveal complex regulatory networks driving therapeutic resistance and immune evasion.
- Tumor microenvironment heterogeneity contributes to immunotherapy failure and treatment relapse.
- Integration of genomics, transcriptomics, and epigenomics provides novel insights into resistance mechanisms.
- A systems-level understanding may guide precision strategies to overcome resistance in RCC.

In recent years, targeted therapies against angiogenic and metabolic signaling pathways have markedly improved outcomes for patients with advanced RCC^[7,8]^. However, the emergence of therapeutic resistance has become a major clinical challenge. A substantial proportion of patients develop primary or acquired resistance within 6–15 months of treatment, resulting in tumor relapse or progression^[9]^. Similarly, although immune checkpoint inhibitors (ICIs) have revolutionized RCC management, their clinical efficacy is hampered by tumor microenvironment heterogeneity and immune tolerance mechanisms, with only 20%–30% of patients achieving durable responses.

Therefore, elucidating the molecular basis of resistance to targeted therapies and immune evasion has become a central challenge in overcoming therapeutic limitations in RCC. Increasing evidence indicates that genetic alterations, epigenetic remodeling, metabolic reprogramming, and modifications of the immune microenvironment collectively contribute to resistance^[10–13]^. However, traditional single-omics approaches are insufficient to comprehensively dissect the dynamic evolution of resistance or to elucidate the complex interplay between gene regulatory networks and phenotypic outcomes.

In this context, multi-omics strategies integrating genomics, transcriptomics, epigenomics, proteomics, and metabolomics provide a powerful framework for systematically deciphering resistance mechanisms^[14]^. These approaches not only identify key driver events but also reveal multi-layered regulatory networks and dynamic biomarkers^[15]^. Moreover, the integration of single-cell sequencing and spatial omics technologies enables precise mapping of tumor heterogeneity and microenvironmental interactions that contribute to resistance^[16]^. Combining multi-omics data with clinical insights holds great promise for developing rational combination therapies, such as targeted-immunotherapy regimens or epigenetic-metabolic interventions, to overcome resistance and advance precision medicine in RCC.

**Figure 1.**
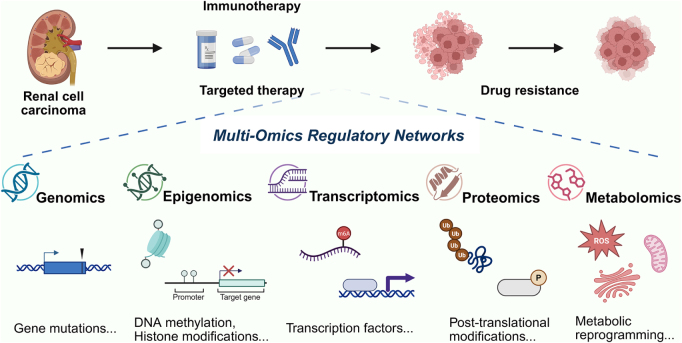
Multi-omics regulatory networks in RCC resistance mechanisms. The diagram depicts the interrelated multi-omics regulatory networks in RCC, illustrating how genomics, epigenomics, transcriptomics, proteomics, and metabolomics converge to drive resistance to targeted therapy and immunotherapy. These omics layers are interconnected, with each influencing the others to regulate tumor progression, immune evasion, and treatment resistance. This figure was created using BioRender (www.Biorender.Com).

This review systematically explores the molecular mechanisms of resistance to targeted therapies and immune evasion in RCC through a multi-omics lens. We highlight recent advances in understanding the roles of genomic mutations, epigenetic regulation, transcriptional control, and post-transcriptional and post-translational modifications (PTMs) in driving resistance to targeted therapies. Subsequently, we delve into multi-omics studies of immune tolerance, examining how tumor genomic alterations, epigenetic remodeling, transcriptional dysregulation, and protein modifications shape the immunosuppressive microenvironment, thereby compromising the efficacy of immunotherapy. By integrating multi-omics data with clinical evidence, this review aims to provide a comprehensive overview of resistance mechanisms in RCC and to lay a theoretical foundation for developing innovative therapeutic strategies (Fig. 1). This review is compliant with the TITAN Guidelines 2025^[17]^.

## Genomics and mutations

### Targeted therapy resistance

**Figure 2.**
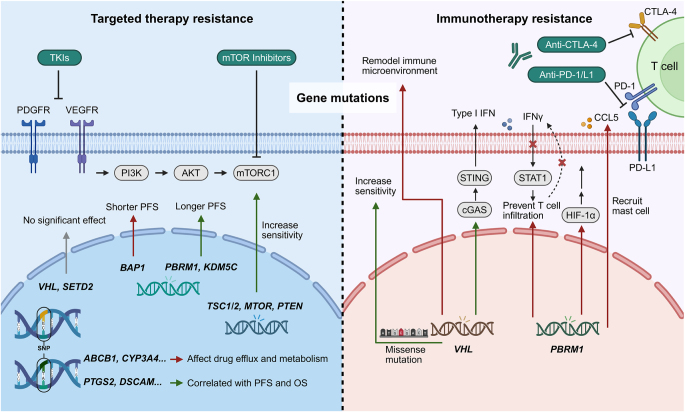
Genomic and mutational alterations contributing to resistance to targeted therapy and immunotherapy. The left panel illustrates representative gene mutations and single nucleotide polymorphisms (SNPs) associated with response or resistance to targeted therapies. The right panel illustrates key mutations involved in immunotherapy outcomes. The role of VHL mutations in immunotherapy response remains controversial, as they may affect efficacy through multiple pathways. This figure was created using BioRender (www.Biorender.Com).

Gene mutations play a pivotal role in the initiation, progression, and treatment response of RCC, influencing the efficacy of targeted therapies through various mechanisms, including alterations in drug targets, activation of compensatory signaling pathways, and enhancement of immune evasion. Among these, inactivation mutations or deletions of the Von Hippel-Lindau (VHL) gene represent the most frequent genetic aberration in RCC, particularly in clear cell RCC (ccRCC), where approximately 90% of cases exhibit VHL alterations^[18]^. These mutations typically result in activation of the hypoxia-inducible factor (HIF) pathway, promoting angiogenesis, tumor proliferation, and metastasis^[19,20]^. Although VHL mutations are strongly associated with RCC pathogenesis, several studies have shown that they do not significantly influence resistance to VEGF or mTOR inhibitors (Fig. 2) ^[21,22]^.

Advances in genomics and multi-omics technologies have enabled the identification of additional genetic alterations associated with resistance, offering new avenues for personalized therapy. Beyond VHL, other frequently mutated genes are also commonly found in RCC include PBRM1, SETD2, KDM5C, PTEN, BAP1, mTOR, and TP53, many of which are involved in chromatin remodeling, DNA repair, and the PI3K/AKT/mTOR pathway^[20,23]^. Similarly, Sato *et al*’s study further elucidated the genetic background of ccRCC using whole-genome, exome, and RNA sequencing technologies^[10,24]^. Understanding these mutations not only elucidates tumor biology but also facilitates mechanistic investigations into therapeutic resistance.

Evidence regarding the clinical relevance of these mutations remains complex. In a study involving 79 patients with mRCC, Kwiatkowski *et al* reported that mutations in TSC1, TSC2, and mTOR correlated with favorable responses to mTOR inhibitors, with a higher frequency of mutations observed in partial responders (Fig. 2)^[22]^.

Similarly, a study analyzing 105 mRCC samples found that patients harboring mutations in the mTOR pathway exhibited better responses to treatment^[25]^. Interestingly, patients with PTEN-deficient tumors were more likely to benefit from mTOR inhibitors, particularly when concurrent mutations in mTOR pathway genes were present. Supporting this observation, Liu *et al* further demonstrated that loss of PTEN enhances dependence on PI3K/AKT/mTOR signaling, increasing the sensitivity of ccRCC cells to mTOR inhibitors. In contrast, mutations in chromatin remodeling genes such as PBRM1, SETD2, BAP1, and KDM5C did not significantly affect mTOR inhibitor efficacy^[26]^.

However, other studies have produced conflicting results. For example, Voss *et al* found no significant association between TSC1, TSC2, or mTOR mutation status and progression-free survival (PFS) in patients treated with everolimus^[27]^. In the RECORD-3 randomized trial, Hsieh *et al* reported that PBRM1 and KDM5C mutations were associated with longer PFS following treatment with everolimus or sunitinib, whereas BAP1 mutations predicted poorer prognosis (Fig. 2). Although high-frequency mutations such as VHL, SETD2, and PTEN were prevalent, they did not demonstrate significant associations with PFS or overall survival (OS) in patients receiving targeted therapy^[21,28]^. Overall, these findings underscore the complex and sometimes contradictory role of genetic mutations in mediating resistance, highlighting the need for further validation in large, well-characterized cohorts.

Beyond point mutations, single nucleotide polymorphisms (SNPs) are another important layer of genetic variation that may influence therapeutic response. SNPs are widespread throughout the genome, and accumulating evidence suggests that they contribute to resistance in mRCC^[29,30]^. Variants in genes such as ABCB1 and ABCG2, which encode ATP-binding cassette (ABC) efflux transporters involved in sunitinib pharmacokinetics, and CYP3A4/CYP3A5, which encode sunitinib-metabolizing enzymes, have been shown to affect drug metabolism, transport, and bioavailability, potentially leading to resistance (Fig. 2)^[31–33]^.

Moreover, SNPs such as rs5275 (PTGS2), rs9582036 (VEGFR1), rs307821 (VEGFR3), rs231775 (CTLA-4), rs28520013 (PDLIM3), and rs2205096 (DSCAM) have been significantly associated with PFS and OS in sunitinib-treated patients (Fig. 2)^[34–39]^. These findings provide a genetic framework for understanding inter-individual variability in therapeutic efficacy. However, the precise molecular mechanisms by which these SNPs influence resistance remain to be elucidated, underscoring the need for further experimental studies and large-scale clinical validation.

### Immunotherapy resistance

The frequent deletion of the VHL gene is a hallmark molecular event in RCC and is considered a key driver of tumor initiation, primarily by disrupting hypoxia-sensing and regulatory pathways^[23]^. Recent studies have further demonstrated that VHL loss also remodels the immune microenvironment (Fig. 2). Specifically, VHL deletion in RCC has been associated with both reduced tumor proliferation and increased infiltration of conventional and regulatory CD4⁺ T cells, as well as the emergence of a glucose-consuming tumor-associated macrophage (TAM) subset with enhanced phagocytic capacity^[40]^. Concurrently, VHL knockout tumors show reduced responsiveness to anti-PD-1 therapy, potentially due to impaired lymphocyte activation.

Conversely, restoration of VHL expression upregulates vascular cell adhesion molecule-1 (VCAM-1), which promotes antitumor immunity through interaction with α4β1 integrins on immune cells^[41]^. However, VHL-deficient tumors may also exhibit enhanced anti-PD-1 efficacy under certain conditions. This has been attributed to HIF-1α/2α-mediated reduction in mitochondrial membrane potential, leading to cytoplasmic leakage of mitochondrial DNA, subsequent activation of the cyclic GMP-AMP synthase-interferon gene-stimulating factor (cGAS-STING) pathway, and induction of type I interferons, which enhance T cell-dependent antitumor responses (Fig. 2)^[42]^. These seemingly contradictory findings may reflect heterogeneity in VHL mutation types.

To address this, recent studies have stratified VHL mutations into two subtypes: truncating mutations (VHL^Trunc^) and missense mutations (VHL^Miss^). Notably, tumors harboring VHL^Miss^ mutations exhibit hyperactivation of cell cycle and NF-κB signaling pathways and show improved sensitivity to immunotherapy (Fig. 2)^[43]^. These findings underscore the importance of stratifying RCC patients based on VHL mutation subtypes to guide personalized treatment strategies.

Similarly, PBRM1 loss-of-function mutations have been shown to impair the efficacy of ICI in RCC. Mechanistically, PBRM1 deficiency disrupts the binding of brahma-related gene 1 (BRG1) to the interferon-γ receptor 2 (IFNGR2) promoter, resulting in reduced expression and secretion of IFNγ (Fig. 2)^[44]^. Additionally, PBRM1 mutations activate HIF-related pathways and upregulate C-C motif chemokine ligand 5 (CCL5), promoting mast cell infiltration and the establishment of an immunosuppressive tumor microenvironment, thereby contributing to tumor progression and poor prognosis (Fig. 2)^[45]^. Therefore, stratifying patients based on PBRM1 mutational status may thus enhance the prediction of immune checkpoint blockade (ICB) response and enable the development of tailored therapeutic approaches^[46]^.

Furthermore, deletion of the chromosome 9p21.3 region has been implicated in both pancreatic metastasis and the classification of a distinct RCC subtype, translocation RCC. This subtype exhibits variable responsiveness to anti-angiogenic therapies but tends to respond more favorably to immunotherapy, potentially due to increased CD8⁺ T cell infiltration^[47,48]^. These insights highlight the necessity of comprehensive mutational profiling in RCC, both for the identification of biologically distinct subtypes and as a foundation for personalized therapeutic decision-making.

## DNA modifications and epigenetics

### Targeted therapy resistance

Epigenetics refers to heritable alterations in gene expression that occur without changes to the underlying DNA sequence^[49,50]^. Epigenetic modifications play a crucial role in the initiation and progression of RCC and are considered key drivers of tumor progression^[51]^. In RCC, common mutations such as PBRM1 and SETD2 are closely linked to defects in chromatin remodeling and epigenetic regulation. These mutations influence the epigenetic landscape of tumor cells, thereby reshaping gene expression patterns, promoting tumor progression, and contributing to therapeutic resistance^[52]^.

Major epigenetic mechanisms include DNA methylation, histone modifications, and chromatin remodeling. Increasing evidence indicates that these modifications are involved in resistance to tyrosine kinase inhibitors (TKIs), such as sunitinib, through regulation of gene transcription, activation of compensatory pathways, modulation of the tumor microenvironment, and enhancement of genomic instability^[11,12,53]^.

**Figure 3.**
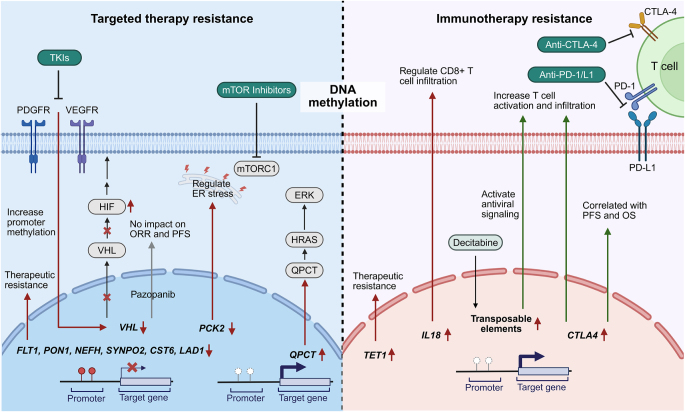
DNA methylation alterations contributing to resistance to targeted therapy and immunotherapy. The left panel shows that hypermethylation or hypomethylation of genes such as VHL, PCK2, and QPCT affects HIF signaling, ER stress, and other regulatory pathways contributing to resistance to targeted therapies. The right panel illustrates the impact of DNA methylation on immunotherapy outcomes, where changes in methylation status of IL18, TET1, and CTLA4 influence immune checkpoint expression and CD8⁺ T cell infiltration, thereby modulating sensitivity or resistance to immune checkpoint blockade. This figure was created using BioRender (www.Biorender.Com).

DNA methylation involves the addition of a methyl group to the fifth carbon of cytosine residues in genomic DNA, catalyzed by DNA methyltransferases (DNMTs), resulting in the formation of 5-methylcytosine (5-mC). This typically occurs in CpG islands within gene promoter regions and represses gene transcription by inhibiting transcription factor binding^[54]^. In ccRCC, VHL gene inactivation is frequently caused by gene mutations or promoter hypermethylation^[55,56]^. Loss of VHL function leads to the accumulation of HIF-1α and HIF-2α, resulting in aberrant activation of HIF signaling pathways (Fig. 3)^[57]^. This activation promotes angiogenesis, metabolic reprogramming, and downstream gene expression changes, creating a favorable environment for resistance to targeted therapies^[11]^.

Stewart *et al* reported that sunitinib treatment significantly increased methylation levels in the VHL promoter region in tumor samples, suggesting an adaptive epigenetic response to therapeutic pressure that may enhance tumor survival via HIF pathway regulation (Fig. 3)^[58]^. Alterations in other key genes such as PBRM1 and CAIX were also observed, further contributing to intratumoral heterogeneity and facilitating resistance mechanisms in mRCC^[59]^. However, in contrast, Choueiri *et al* found no significant correlation between VHL mutation or methylation status and objective response rate or PFS in ccRCC patients treated with pazopanib (Fig. 3)^[60]^, highlighting the complexity of epigenetic regulation. Other epigenetically regulated genes have also been implicated in TKI resistance. Zhao *et al* showed that RCC patients with low expression of glutaminyl peptide cyclotransferase (QPCT) exhibited significantly improved PFS following sunitinib treatment^[61]^. Mechanistically, QPCT promoter hypomethylation enhances NF-κB (p65) binding and upregulates QPCT transcription. Elevated QPCT stabilizes HRAS by preventing its ubiquitin-mediated degradation, which in turn activates the ERK signaling pathway and promotes resistance (Fig. 3)^[61,62]^. Similarly, hypermethylation of the FLT1 promoter suppresses its expression, thereby impairing the anti-proliferative effects of sunitinib and axitinib, whereas FLT1 demethylation restores its expression and enhances treatment efficacy^[63]^. The metabolic gene PCK2 is also downregulated in RCC due to promoter hypermethylation. Restoring PCK2 expression through the demethylating agent 5-AZA or CRISPR/dCas9-mediated targeted demethylation sensitizes RCC cells to sunitinib by promoting endoplasmic reticulum (ER) stress (Fig. 3)^[64]^. Additionally, PON1 promoter hypermethylation has been linked to increased proliferation, migration, and invasion in sunitinib-resistant RCC cells. Treatment with 5-AZA and histone deacetylase (HDAC) inhibitors restores PON1 expression and improves sunitinib sensitivity^[65]^. Furthermore, methylation of other genes, including NEFH, SYNPO2, CST6, and LAD1, has been linked to resistance to anti-angiogenic therapy (Fig. 3). Specifically, NEFH and SYNPO2 methylation are correlated with poor therapeutic response, whereas hypermethylation of CST6 and LAD1 is associated with shortened PFS and reduced OS in ccRCC patients^[66–68]^. Collectively, these findings highlight the central role of epigenetic dysregulation in driving resistance to targeted therapies and underscore the clinical potential of epigenetic biomarkers and epigenetic-based therapeutic strategies for personalized treatment of RCC patients.

### Immunotherapy resistance

#### DNA methylation

Growing evidence indicates that aberrant DNA methylation plays a critical role in the progression of RCC by modulating gene expression patterns. For example, Deng *et al* reported that hypomethylation of the IL18 promoter contributes to its overexpression in RCC. This epigenetic change was also associated with downregulation of immune checkpoint molecules and increased infiltration of CD8⁺ T cells, suggesting its potential as a predictive biomarker for immunotherapy responsiveness (Fig. 3)^[69]^.

In another study, Lu *et al* identified a subgroup of ccRCC tumors with high levels of enhancer demethylation (termed TED) through Illumina EPIC profiling of patient samples treated with ipilimumab plus nivolumab or sunitinib. This TED subgroup displayed poor prognosis and resistance to ICI therapy. Mechanistically, TED was linked to demethylation of the TET1 promoter and activation of specific downstream regulators, which together serve as predictors of immunotherapy resistance (Fig. 3)^[70]^.

Based on the study of DNA methylation modification patterns in RCC, Bai *et al* further classified RCC into three distinct DNA methylation modification patterns, C1, C2, and C3, corresponding to immune-desert, immune-excluded, and immune-inflamed phenotypes, respectively. This classification provides an innovative complement to traditional subtyping methods and supports the stratification of patients for precision immunotherapy^[71]^.

From a therapeutic perspective, targeting DNA methylation has shown promising potential. In a study by WK and colleagues, treatment with the DNMT inhibitor decitabine enhanced transposable element expression, activated innate antiviral signaling, and promoted T cell infiltration and activation, thereby enhancing ICI efficacy in RCC models (Fig. 3)^[72]^.

Given the prominent role of DNA methylation in RCC, methylation-driven gene expression models have shown prognostic potential. Li *et al* constructed a risk model incorporating eight differentially methylated genes, stratifying patients into high- and low-risk groups, and providing a framework to assess immunotherapy efficacy based on risk profiles^[73]^. Among immune checkpoints, CTLA4 has emerged as both a therapeutic target and a biomarker of immunotherapy response. Hypomethylation of the CTLA4 promoter has been shown to correlate with elevated mRNA expression and increased immune cell infiltration, particularly by CD8⁺ T cells (Fig. 3, Table 1). In a multicenter RCC-ICB cohort, CTLA4 promoter hypomethylation was associated with improved PFS and OS, supporting its potential as an independent predictor of immunotherapy benefit and a biomarker for response monitoring (Fig. 3)^[74]^.

**Table 1:** Expression regulation of immune checkpoint molecules in immunotherapy resistance.

Immune checkpoint molecule	Regulatory mechanism	Regulatory type	Resistance mechanism	Related studies	Reference PMID
PD-L1	Transcription factor activates PD-L1 transcription	Transcriptional regulation	Enhances PD-L1 expression, suppresses T-cell activity, and promotes immune evasion	PYK2 phosphorylates TAZ, enhancing its stability and promoting PD-L1 transcription	33 837 850
	Transcription factor activates PD-L1 transcription	Transcriptional regulation	Inhibits tumor-infiltrating CD8+ T-cell activity, mediates immune evasion	TFEB directly binds to the PD-L1 promoter, promoting its transcription	31 383 732
	Transcription factor activates PD-L1 transcription	Transcriptional regulation	Promotes immune evasion and is associated with Sunitinib resistance	TFE3 binds to the PD-L1 promoter, enhancing its expression	33 145 941
	Transcription factor activates PD-L1 transcription	Transcriptional regulation	Promotes tumor immune evasion	HIF-2α binds to the PD-L1 promoter, upregulating its expression	34 789 838
	m6A methylation regulates PD-L1 mRNA stability	Post-transcriptional modification	Enhances PD-L1 expression, suppresses T-cell activity	METTL3 mediates m6A methylation of PD-L1 mRNA, regulating its stability	35 197 058
	miRNA regulation of PD-L1 expression	Post-transcriptional modification	Regulates PD-L1 expression through miRNAs, affecting immune evasion	EBV miR-BART11 and BART17-3p inhibit FOXP1 and PBRM1, enhancing PD-L1 expression	35 165 282
	lncRNA regulation of PD-L1 expression	Post-transcriptional modification	Regulates PD-L1 expression through lncRNAs, affecting immune evasion	lncRNA SNHG1 regulates PD-L1 expression via the miR-129-3p/STAT3 axis	33 739 543
	lncRNA regulation of PD-L1 expression	Post-transcriptional modification	Linc00926 promotes PDL1 expression on the surface of RCC cells	Linc00926 promotes PDL1 expression on the surface of RCC cells	37 866 544
	Phosphorylation modification regulates PD-L1 expression	Post-transcriptional modification	Lack of glucose supply induces PDL1 expression	Glycolysis regulates PDL1 expression by modulating overall EGFR/ERK/c-Jun phosphorylation levels	33 462 221
	Phosphorylation modification regulates PD-L1 expression	Post-transcriptional modification	DCLK1 phosphorylation promotes PDL1 expression	DCLK1 phosphorylation promotes PDL1 expression	34 830 884
	Ubiquitination modification regulates PD-L1 expression	Post-transcriptional modification	GSK3β mediates phosphorylation-dependent proteasomal degradation of PDL1	GSK3β binds to non-glycosylated PDL1 and leads to phosphorylation-dependent proteasomal degradation of PDL1 via β-TrCP	27 572 267
	Ubiquitination modification regulates PD-L1 expression	Post-transcriptional modification	Inhibits PD-L1 expression, enhances immune response	STUB1 mediates PD-L1 degradation through ubiquitination	28 813 410
	Deubiquitination modification regulates PD-L1 expression	Post-transcriptional modification	Enhances PD-L1 expression, suppresses T-cell activity	USP22 stabilizes PD-L1 and CSN5 through deubiquitination, promoting PD-L1 expression	32 665 011
	Deubiquitination modification regulates PD-L1 expression	Post-transcriptional modification	Enhances PD-L1 expression, suppresses T-cell activity	USP9X stabilizes PDL1 by deubiquitination and enhances its expression in tumor cells	29 992 764
	Lactylation modification regulates PD-L1 expression	Post-transcriptional modification	Lactylation promotes PD-L1 expression, suppressing T-cell activity	Lactylation of H3K18 activates PD-L1 transcription	39 137 401
	Acetylation modification regulates PD-L1 expression	Post-transcriptional modification	Acetylation affects PD-L1 nuclear translocation, suppressing immune response	p300 mediates PD-L1 acetylation, HDAC2 mediates deacetylation, regulating PD-L1 nuclear translocation	32 839 551
PD-1	Ubiquitination regulates PD-1 degradation	Post-transcriptional modification	Inhibits PD-1 expression, enhances immune response	FBXO38 mediates PD-1 degradation through ubiquitination	30 487 606
	Phosphorylation regulates PD-1 function	Post-transcriptional modification	Phosphorylation of PD-1 promotes SHP2 signaling, suppressing T-cell activity	PD-1 Y248 phosphorylation promotes SHP2 recruitment, inhibiting T-cell activity	32 184 441
	Deubiquitination regulates PD-1 expression	Post-transcriptional modification	Enhances PD-1 expression, suppresses T-cell activity	USP9X stabilizes PD-1 through deubiquitination	25 200 027
CTLA4	DNA methylation regulates CTLA4 expression	DNA modifications	CTLA4 promoter hypomethylation enhances its expression, promoting immune evasion	CTLA4 promoter hypomethylation correlates with mRNA expression and CD8+ T-cell infiltration	34 446 578

#### Histone modifications

Histone modifications also contribute significantly to the regulation of immune responses in RCC. Major histocompatibility complex class I (MHC-I) antigens are critical for tumor immune recognition, and their downregulation is a known mechanism of immune evasion. α-Ketoglutarate (αKG), a tricarboxylic acid cycle intermediate, has been shown to enhance MHC-I expression in RCC by modulating broad-spectrum histone demethylation and upregulating β2-microglobulin (B2M). Notably, transcriptional upregulation of B2M was dependent on demethylation at the H3K4me1 histone mark at its promoter^[75]^.

Importantly, combined treatment with αKG and PD-1 blockade in murine models led to improved immunotherapy efficacy and prolonged survival, offering a potential synergistic therapeutic strategy for RCC. Additionally, histone deacetylase 3 (HDAC3) has been implicated in modulating immune responses. While HDAC3 suppresses RCC cell proliferation, its activity also promotes T cell activation and elevates IFN-γ production, thereby enhancing T cell-mediated tumor cytotoxicity^[76]^.

Together, these findings demonstrate that histone modifications can reshape the tumor immune microenvironment and provide new opportunities to improve the efficacy of immunotherapy in RCC.

## Transcriptional modification

### Targeted therapy resistance

Transcriptional regulation refers to the modulation of gene activity at the transcriptional level, governed by transcription factors and various co-regulators that respond to intracellular and extracellular cues^[77]^. Signals such as cell cycle progression, environmental fluctuations, nutrient availability, and cellular stress influence the initiation, elongation, and termination of gene transcription^[78]^.

The core mechanisms of transcriptional regulation include: (1) transcription factors, which bind to specific DNA sequences (promoters/enhancers) to recruit or inhibit RNA polymerase, thus initiating or repressing transcription^[79]^; (2) epigenetic modifications, such as DNA methylation and histone acetylation/methylation, which alter chromatin accessibility to the transcriptional machinery^[80]^; and (3) transcriptional initiation complexes, where multiple transcription factors assemble at promoter regions to facilitate polymerase recruitment and transcription start^[81]^.

Transcriptional control operates through positive and negative regulation. In positive regulation, proteins bind promoters to activate transcription, typically by enhancing RNA polymerase recruitment and facilitating initiation and elongation^[82,83]^. In negative regulation, proteins repress transcription by binding regulatory elements, altering chromatin dynamics, or inducing transcriptional pausing or termination^[84,85]^. This intricate regulatory network allows cells to adapt their gene expression programs to specific developmental stages, environmental changes, and metabolic demands. However, dysregulated transcriptional control can profoundly alter cellular homeostasis and drive drug resistance in RCC.

**Figure 4.**
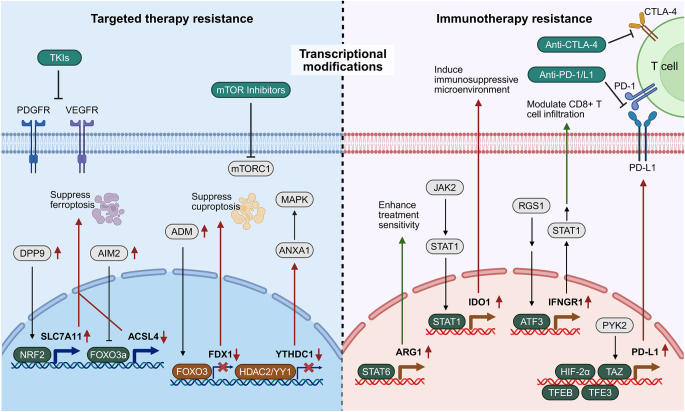
Transcriptional alterations contributing to resistance to targeted therapy and immunotherapy. The left panel illustrates key transcriptional regulators such as NRF2 and FOXO3, and their downstream targets including SLC7A11 and FDX1, which modulate ferroptosis, cuproptosis, and other regulatory pathways contributing to resistance to targeted therapies. The right panel summarizes transcriptional mechanisms of immune resistance, where upregulation of ARG1, IDO1, IFNGR1, and PD-L1 driven by transcription factors such as STAT1, STAT6, ATF3, and TAZ suppresses CD8⁺ T cell activity and promotes an immunosuppressive tumor microenvironment. This figure was created using BioRender (www.Biorender.Com).

Excessive activation of NRF2 is frequently observed in multiple cancer types and has been implicated in tumorigenesis, metastasis, and therapy resistance^[86,87]^. Chang *et al* identified dipeptidyl peptidase 9 (DPP9) as a regulator of the KEAP1–NRF2 axis, where DPP9 binds KEAP1 via a conserved ESGE motif and inhibits ferroptosis, promoting sorafenib resistance in ccRCC (Fig. 4)^[88]^. Similarly, Wang *et al* revealed that AIM2 drives RCC progression and sunitinib resistance in an inflammasome-independent manner by promoting FOXO3a phosphorylation and proteasomal degradation, thereby reducing ACSL4 transcription and inhibiting ferroptosis (Fig. 4)^[89]^. Furthermore, NUPR1 silencing reverses sorafenib resistance^[90]^, while SEC14L3 downregulation enhances sunitinib sensitivity and inhibits ccRCC progression by disrupting the SEC14L3/RPS3/NF-κB feedback loop^[91]^. Thus, targeting SEC14L3 may provide a novel therapeutic strategy. YTHDC1, an m6A reader, inhibits ccRCC progression via the ANXA1/MAPK axis and regulates TKI sensitivity (Fig. 4)^[92]^. Additionally, HDAC2 inhibitors restore drug response by disrupting the YY1/HDAC2 transcriptional complex.

The newly identified form of cell death, cuproptosis, has also been implicated the antitumor activity and drug resistance^[93,94]^. Wang *et al* discovered that adrenomedullin (ADM) upregulation activates p38/MAPK signaling and forkhead box O3 (FOXO3) nuclear translocation, which suppresses FDX1 transcription and apoptosis, leading to sunitinib resistance (Fig. 4)^[95]^. In addition, Ruan *et al* identified Y-box binding protein 1 (YB1) and EphA2, a receptor tyrosine kinase (RTK) family member, as drivers of sunitinib resistance in mRCC^[96]^.

Emerging evidence highlights the contribution of non-coding RNAs to TKI resistance. CircPTPN12 promotes RCC cell proliferation, migration, and invasion while facilitating sunitinib resistance by interacting with hnRNPM to stabilize IL-6 mRNA, leading to STAT3 activation (Fig. 4)^[97]^. Likewise, SIX1 induces metastasis and resistance through focal adhesion (FA) signaling. Mechanistically, integrin subunit β1 (ITGB1), an upstream gene of FA signaling, serves as a direct transcriptional target of SIX1. Furthermore, DHX9, a critical SIX1 cofactor, mediates SIX1-driven ITGB1 transcription. Silencing SIX1/DHX9 disrupts DHX9 nuclear translocation and its binding to the ITGB1 promoter, effectively reducing ITGB1 expression and attenuating TKI resistance^[98]^.

He *et al* elucidated the role of CHD1L in sunitinib resistance, demonstrating that SIRT7 physically interacts with CHD1L and mediates its deacetylation, with SIRT7 exerting its pro-tumorigenic effects in a CHD1L-dependent manner. Accumulated CHD1L amplifies HIF-2α-driven transcriptional programs by directly binding HIF-2α, while CHD1L overexpression sustains VEGFA-mediated resistance, an effect that can be reversed by CHD1L inhibition^[99]^. He *et al* discovered that sunitinib treatment upregulates lncRNA ECVSR, which enhances ERβ mRNA stability and drives HIF-2α transcription, promoting resistance. Combining sunitinib with the anti-estrogen PHTPP significantly improves efficacy and reduces vasculogenic mimicry formation^[100]^. Similarly, Gu *et al* reported that targeting the ERβ/ANGPT-2/Tie-2 signaling pathway with the FDA-approved anti-estrogen Faslodex enhances sunitinib response and may provide a novel combination therapy for advanced ccRCC^[101]^. Wang *et al* identified that inhibition of RBCK1 suppresses AKT and MAPK signaling, reversing sunitinib resistance. Mechanistically, RBCK1 promotes ANKRD35 degradation, which destabilizes MITD1 via SUMO2 binding, forming the RBCK1–ANKRD35–MITD1–ANXA1 axis that regulates AKT/ERK phosphorylation and mediates drug resistance^[102]^. Zhu *et al* demonstrated that ZHX2 promotes tumor growth and sunitinib resistance by upregulating VEGF, activating the MEK/ERK1/2 pathway, and inducing protective autophagy, highlighting a potential therapeutic target in advanced ccRCC^[103]^. Guo *et al* showed that TFE3-driven PD-L1 upregulation contributes to sunitinib resistance via immune evasion mechanisms^[104]^. Yao *et al* reported that sunitinib downregulates GATA1, reducing its binding to the MIR885 promoter and increasing miR-885-5p activity, which directly upregulates PLIN3 expression, enhances lipid droplet formation, and diminishes drug sensitivity^[105]^. Liu *et al* revealed that the lncRNA SNHG12/SP1/CDCA3 axis promotes RCC progression and sunitinib resistance, suggesting a potential therapeutic target for drug-refractory tumors^[106]^. Shi *et al* reported that the TR4 nuclear receptor regulates lncTASR transcription, stabilizing AXL mRNA and enhancing AXL protein expression, thereby promoting sunitinib resistance^[107]^.

Resistance to bevacizumab poses a significant challenge in ccRCC, and the underlying epigenetic regulators of bevacizumab sensitivity remain largely unexplored^[108]^. Cheng *et al* demonstrated that UCHL1 mediates KDM4B deubiquitination, stabilizing KDM4B, which binds the VEGFA promoter and removes the inhibitory H3K9me3 modification. Inhibition of UCHL1 using the small-molecule inhibitor 6RK73 significantly suppressed ccRCC progression and enhanced bevacizumab sensitivity^[109]^. The findings reported by Schiavoni *et al* demonstrate that downregulation of PON2 can elicit a reduction in the proliferation and migration of ccRCC cells, along with an enhancement in cellular sensitivity to chemotherapy^[110]^. Lu *et al* identified the Solute Carrier Family 27 Member 3 (SLC27A3), a lipid transporter highly expressed in lipid-rich ccRCC, as a driver of pazopanib resistance Elevated SLC27A3 enhances long-chain fatty acyl-CoA production, fueling mitochondrial β-oxidation and generating excess reactive oxygen species (ROS), which activate mitophagy. A resulting ROS/mitophagy feedback loop maintains ROS homeostasis but consumes CPT1A, inhibiting fatty acid oxidation and forcing lipid storage in droplets, thereby mediating resistance^[111]^. Xiao *et al* reported that RNF7 promotes STAT3 signaling by ubiquitinating SOCS1, which suppresses apoptosis, enhances glycolysis, and reduces sunitinib sensitivity^[112]^. Xu *et al* experimentally demonstrated that the traditional Chinese medicine Sanhuang decoction restores axitinib sensitivity and inhibits tumor growth in resistant models by modulating immune infiltration and ADAMTS18 expression^[113]^. In addition, Yu *et al* described a regulatory axis whereby BMP8A promotes Nrf2 phosphorylation and activates TRIM24 to enhance survival and drug resistance in ccRCC^[114]^.

Cowman *et al* utilized single-cell sequencing to reveal that HIF-1α is predominantly expressed in TAMs, with elevated levels of HIF-1α in TAMs showing a significant correlation with resistance to anti-angiogenic therapy^[115]^. Consistent with this, ccRCC is characterized by high angiogenic activity and robust vasculogenesis^[116]^. Marona *et al* found that both sunitinib and sorafenib resistance can lead to c-Met/IRAK1 activation and MCPIP1 downregulation, which collectively enhance tumor metastasis, likely due to increased vascularization in resistant tumors^[117]^. Wang *et al* further revealed that UBB regulates VEGFA transcription by directly interacting with specificity protein 1 (SP1), and a disrupted UBB/VEGFA ratio drives pazopanib resistance in ccRCC^[118]^. In a genomic and transcriptomic analysis, Dizman *et al* identified frequent alterations in VHL (64%), PBRM1 (38%), SETD2 (24%), KDM5C (17%), and TERT (12%), underscoring their potential roles as predictors of TKI response^[119]^. Wang *et al* observed that gankyrin overexpression in ccRCC cell promotes proliferation, invasion, migration, and pazopanib resistance while suppressing apoptosis. Mechanistically, gankyrin recruits STAT3, which transcriptionally activates CCL24. The elevated CCL24 then signals through CCR3 to further enhance gankyrin expression and STAT3 activation, establishing a gankyrin/STAT3/CCL24/CCR3 positive feedback loop. Disrupting this autocrine circuit may represent a promising strategy for treating advanced ccRCC^[120]^.

Interestingly, studies identifying resistance mechanisms also reveal potential sensitization strategies. For instance, Hu *et al* found that the AKT activator SC79 can reverse the inhibitory effect of TAF1D knockdown in ccRCC cells. Notably, TAF1D knockdown sensitizes ccRCC cells to sunitinib and enhances sunitinib-induced tumor cell suppression^[121]^. Cabozantinib, a c-Met/RTK inhibitor, has been approved for the treatment of advanced RCC^[122]^. In a recent study, LaRawat *et al* reported that combining cabozantinib with honokiol synergistically suppresses RCC cell growth by increasing ROS production, suggesting that honokiol may enhance cabozantinib efficacy and represents a potential therapeutic option for overcoming resistance^[123]^.

### Immunotherapy resistance

#### Transcriptional regulation of immune checkpoint molecules

The regulation of the transcriptional expression of immune checkpoint molecules has been an area of intense research interest in RCC. It has been found that the PDZ-binding motif (TAZ) protein is able to activate PD-L1 transcription by binding to its promoter region, while the upstream kinase proline-rich/Ca-activated tyrosine kinase 2 (PYK2) phosphorylates TAZ, thereby stabilizing the protein and enhancing its activity (Fig. 4)^[124]^. Thus, inhibition of the PYK2/TAZ/PDL1 signaling axis may enhance the response of RCC cells to immunotherapy. Transcription factors EB (TFEB) and E3 (TFE3) also play critical roles in immune escape in RCC (Fig. 4). TFEB directly binds to the PD-L1 promoter, promoting its transcription and suppressing tumor-infiltrating CD8+ T cells activity, thereby facilitating immune escape^[125]^. Similarly, TFE3, a key oncogene for RCC proliferation, enhances PD-L1 transcription by interacting with its promoter, leading to both immune escape and sunitinib resistance^[104]^. However, compared with ccRCC and papillary RCC, significantly fewer M1-macrophages (0.8%) were observed in TFE3-rearranged renal cell carcinoma (rRCC; *P* < 0.05), predicting a colder tumor immune microenvironment. However, the increased proportion of PD-L1⁺ tumor cells in rRCC paradoxically enhances the therapeutic benefit of ICI therapy^[126]^. In addition, HIF-2α was significantly expressed in RCC and promoted tumor immune escape by binding to the hypoxia-responsive element of the PD-L1 promoter and upregulating PD-L1 transcription (Fig. 4)^[127]^. Together, these mechanisms reveal a complex network of immune checkpoint molecules regulation in RCC and its role in tumor progression (Table 1).

#### Transcriptional regulation of cytokines and chemokines

Cytokines and chemokines play critical roles in modulating immune cell infiltration, activation, and tumor immune responses in RCC, thereby contributing to the development of immunotherapy resistance. Several immunosuppressive cytokines, such as transforming growth factor-β (TGF-β), interleukin-10 (IL-10), interleukin-6 (IL-6), VEGF, interleukin-23 (IL-23), and interleukin-17 (IL-17), have been demonstrated to inhibit antitumor immunity directly or indirectly and thus might affect the effect of immunotherapy^[128–133]^. Transcriptional studies targeting these factors are expected to reveal new mechanisms of immunotherapy resistance. For example, STAT3 and NF-κB regulate the transcriptional expression of IL-10 and directly bind to the IL-6 promoter and promote its transcription^[134–136]^. Meanwhile, STAT3 also directlyactivates IL23 transcription and cooperates with interferon regulatory factor 4 (IRF4) to induce IL-23-dependent downstream gene expression^[137,138]^. In addition, the transcription factor RORγt (RORC), a master regulator of Th17 cell differentiation, directly binds to the IL-17A promoter to promote the transcription of IL-17A and simultaneously upregulates IL-23 receptor expression, thus enhancing the activity of the IL-23 signaling pathway^[133]^. These studies provide new insights for deeper elucidation of immunotherapy resistance mechanisms.

Some chemokines also exert potent immunomodulatory effects. For example, CXCL12 is able to recruit regulatory T cells (Tregs) and thus shape the suppressive immune microenvironment, whereas its receptor, C-X-C chemokine receptor type 4 (CXCR4), is significantly expressed in RCC, and its transcription is tightly controlled by HIF-1α, which directly binds the CXCR4 promoter. Therapeutic strategies targeting CXCR4 markedly reduced FOXP3-TSDR demethylation and downregulated DNMT1 and FOXP3, thereby inhibiting Tregs function and potentially enhancing the efficacy of immunotherapy^[139,140]^. In a study on the mechanism of action of cabozantinib, researchers found that the drug induces the expression of neutrophil-associated chemokines (e.g., CCL11 and CXCL12) and T-cell-associated chemokines (e.g., CCL8 and CX3CL1), potentially driving long-term antitumor T-cell responses. These findings provide a theoretical rationale for combining cabozantinib with T-cell-targeted therapies or ICI agents^[141]^. Moreover, Regulator of G-protein signaling 1 (RGS1) promotes the binding of activating transcription factor 3 (ATF3) to the interferon gamma receptor 1 (IFNGR1) promoter, thereby upregulating STAT1 and IFNγ-inducible genes such as CXCL9 and MHC-I. This modulation of antigen presentation and CD8⁺ T-cell infiltration identifies a novel pathway contributing to RCC immunotherapy resistance (Fig. 4)^[142]^.

#### Transcriptional regulation of metabolism-related pathways

Given the critical role of metabolic reprogramming in RCC, alterations in the transcriptional regulation of metabolism-related factors substantially affect the tumor microenvironment and contribute to immune escape and therapeutic resistance^[143,144]^. In a clinical study of targeted therapy combined with immunotherapy in RCC, Xue and colleagues found that treatment-sensitive patients exhibited significantly higher expression of arginase 1 (ARG1)^[145]^. Mechanistically, ARG1 transcription is predominantly regulated by the signal transducer and activator of transcription 6 (STAT6) pathway, which directly binds to the ARG1 promoter and enhances its expression (Fig. 4)^[146]^. Another key immune-metabolic factor, indoleamine 2,3-dioxygenase 1 (IDO1), plays a pivotal role in immune tolerance and tumor immune evasion. IDO1 expression leads to T cell and NK cell inactivation while simultaneously promoting the recruitment and functional activation of Tregs and myeloid-derived suppressor cells (MDSCs), collectively establishing an immunosuppressive tumor microenvironment^[147–149]^. Importantly, studies have shown that the IDO1 transcription is primarily regulated by the JAK2-STAT1 signaling pathway, which exhibits enhanced stability upon activation by upstream signals. This cascade promotes increased IDO1 expression, thereby attenuating the efficacy of anti-PD-1 therapy in RCC (Fig. 4)^[150]^.

#### Immune cell regulation in the tumor microenvironment

The infiltration and functional activation of suppressive immune cells represent major mechanisms driving immunotherapy resistance in RCC^[151]^. Single-cell and spatial transcriptome RNA sequencing results demonstrated that Tregs are enriched in the RCC microenvironment, where they potently suppress antitumor immune responses. The differentiation and immunosuppressive function of Tregs are tightly controlled by the transcription factor FoxP3^[152,153]^. Mechanistically, nuclear FoxP3 directly binds DNA-associated proteins generated during Treg activation or differentiation, thereby stabilizing its association with chromatin and dynamically regulating Treg function according to the immune context^[154]^. Interestingly, recent studies found that FoxP3 is also highly expressed in RCC cells and correlates with poor prognosis, which may be caused by BAP1 or SEDT2 mutations, suggesting that FoxP3 could also function as a potential oncogene during RCC progression^[155]^.

The differentiation and function of MDSCs are also regulated by transcription factors. CCAAT/Enhancer Binding Protein ß (C/EBPß) controls emergency myelopoiesis, and dysregulation of C/EBPß activity leads to abnormal myelopoiesis and pronounced MDSC expansion^[156,157]^. Similarly, STAT3 coordinates both MDSCs and the acquisition of immunosuppressive properties. Inhibition of STAT3 induces apoptosis in MDSC, increases upregulated molecules involved in antigen processing and presentation, and suppresses immunosuppressive factors expression, thus diminishing the MDSC-mediated suppression of antitumor immunity^[158]^. Beyond its role in MDSCs, STAT3 critically regulates CD8⁺ T-cell differentiation. Studies have shown that STAT3 enhances the T_ex_^term^ transcriptional program by activating T_ex_^term^-associated genes and repressing the expression of T_ex_^prog^ genes through epigenetic mechanisms. In addition, STAT3 synergizes with basic leucine zipper ATF-like transcription factor (BATF) and IRF4 to mediate T_ex_^term^ cell differentiation^[159]^. These findings indicate that STAT3 signaling plays a critical role in the terminal differentiation of CD8+ T cells, offering a theoretical basis for developing novel immunotherapies targeting STAT3-driven immune dysfunction.

## Post-transcriptional modifications

**Figure 5.**
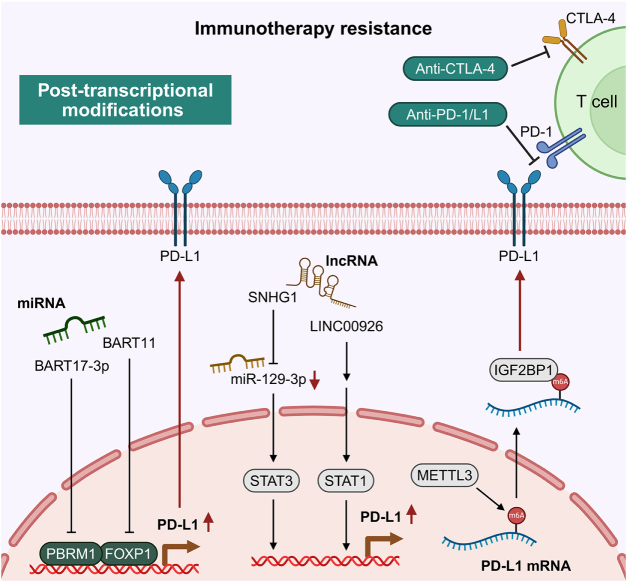
Post-transcriptional modifications associated with immunotherapy resistance. Post-transcriptional mechanisms, including non-coding RNAs (miRNAs and lncRNAs) and m6A RNA methylation, regulate PD-L1 expression and mediate immunotherapy resistance in RCC. This figure was created using BioRender (www.Biorender.Com).

Post-transcriptional modifications refer to a series of chemical processes that alter RNA molecules after transcription is complete, including 5’ capping, 3’ polyadenylation, RNA splicing, m6A methylation, RNA editing, and non-coding RNA-mediated regulation. These modifications finely tune RNA stability, processing, and function, representing crucial steps for accurate gene expression and the production of functional RNA molecules. Recent research has increasingly revealed that post-transcriptional modifications play a central role in resistance to both targeted therapy and immunotherapy in RCC by regulating the expression of immune-related genes (such as PD-L1), shaping the tumor microenvironment, and mediating immune escape (Fig. 5). Therefore, elucidating the molecular mechanisms underlying these RNA modifications is essential for uncovering resistance pathways and developing novel therapeutic strategies for RCC.

For a detailed description of the role of post-transcriptional modifications in mediating RCC drug resistance, as illustrated in Figure 5 and Table 1, please refer to the description in Supplemental Digital Content File S1, available at: http://links.lww.com/JS9/G864.

## Post-translational modifications

PTMs encompass a range of covalent alterations to amino acid residues after protein synthesis on ribosomes^[160]^. Although PTMs do not alter the primary amino acid sequence, they profoundly influence protein conformation, biological functions, subcellular localization, and molecular interactions^[161]^. Essentially, PTMs represent “post-processing” mechanisms that enable proteins to execute precise and dynamic functions within diverse cellular contexts^[162]^. The spectrum of PTMs is extensive, encompassing acetylation^[163]^, methylation^[164]^, ubiquitination^[165]^, phosphorylation^[166]^, glycosylation^[167]^, and acylation^[168]^, among others.

By modifying specific amino acid residues, PTMs regulate protein–DNA, protein–RNA, and protein–protein interactions, thereby modulating gene expression and cellular responses^[169]^. Moreover, PTMs orchestrate signal transduction pathways: following environmental stimuli, many signaling molecules undergo PTM-dependent conformational and functional changes, transmitting cues to downstream effectors and coordinating adaptive responses^[170]^. In addition, PTMs influence protein degradation, subcellular trafficking, and other essential processes, maintaining normal cellular homeostasis^[171]^.

**Figure 6.**
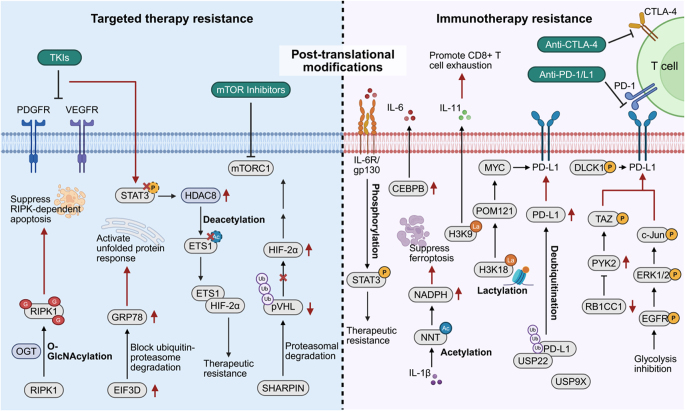
Post-translational modifications contributing to resistance to targeted therapy and immunotherapy. The left panel summarizes post-translational mechanisms involved in targeted therapy resistance, including O-GlcNAcylation, deacetylation, phosphorylation, and ubiquitination, which affect apoptosis, unfolded protein response, and HIF signaling. The right panel illustrates post-translational events contributing to immunotherapy resistance, including acetylation, lactylation, phosphorylation, and deubiquitination, which modulate ferroptosis, immune checkpoint stability, and T cell exhaustion. This figure was created using BioRender (www.Biorender.Com).

For a detailed description of the role of PTMs in mediating RCC resistance, as illustrated in Figure 6 and Table 1, please refer to the description in Supplemental Digital Content File S1, available at: http://links.lww.com/JS9/G864.

## Conclusion and future directions

Recent advances in multi-omics technologies have provided unprecedented insights into the complex mechanisms underlying resistance to targeted therapies and immune tolerance in RCC^[172]^. By integrating data from genomics, epigenomics, transcriptomics, proteomics, and metabolomics, researchers have begun to unravel the dynamic, multi-layered evolution of drug resistance and immune evasion. These findings not only deepen our understanding of RCC biology but also hold significant promise for clinical translation.

From a clinical perspective, multi-omics approaches have enabled the identification of robust biomarkers for predicting therapeutic response and resistance, facilitating patient stratification and personalized treatment strategies. For instance, genomic and epigenomic profiling of VHL and PBRM1 mutations, PD-L1 promoter methylation status, and immune-related lncRNA signatures offers valuable tools for anticipating outcomes to ICIs^[173–175]^. Similarly, transcriptomic and proteomic signatures associated with metabolic reprogramming or PTMs may guide the selection of combination therapies, such as mTOR inhibitors with epigenetic modulators or ICIs with metabolic interventions^[75,176]^.

The integration of liquid biopsies and longitudinal multi-omics profiling into clinical workflows represents a transformative approach for dynamic monitoring of treatment response and resistance evolution^[177,178]^. Such strategies could enable real-time adjustment of therapeutic regimens, minimize toxicity and maximize efficacy. Furthermore, the discovery of novel therapeutic targets, such as lactylation-regulated immune suppression, ubiquitination-mediated PD-L1 stability, and transcriptional networks controlling cytokine secretion, paves the way for developing next-generation drugs and rational combination therapies^[179,180]^.

To accelerate clinical translation, future efforts should focus on validating these multi-omics-derived biomarkers and targets in large, prospective clinical cohorts. Collaborative initiatives integrating multi-omics data with electronic health records and real-world evidence will be essential for refining predictive models and advancing precision oncology. Ultimately, the convergence of multi-omics insights with clinical practice promises to redefine therapeutic paradigms in RCC, overcoming resistance and improving survival outcomes for patients.

In conclusion, the in-depth application of multi-omics technologies will drive a paradigm shift in the study of resistance mechanisms in RCC, laying a solid foundation for precision medicine in the era of individualized therapy. Through interdisciplinary collaboration and technological innovation, we are poised to achieve transformative advances in the treatment of RCC.

## Data Availability

The data that support the findings of this study are available from the corresponding author upon reasonable request

## References

[1] Bray F Laversanne M Sung H. Global cancer statistics 2022: GLOBOCAN estimates of incidence and mortality worldwide for 36 cancers in 185 countries. CA Cancer J Clin 2024;74:229–63. 38572751 10.3322/caac.21834

[2] Tsivian M Moreira DM Caso JR. Cigarette smoking is associated with advanced renal cell carcinoma. J Clin Oncol 2011;29:2027–31. 21502558 10.1200/JCO.2010.30.9484

[3] Venkatesh N Martini A McQuade JL. Obesity and renal cell carcinoma: biological mechanisms and perspectives. Semin Cancer Biol 2023;94:21–33. 37286114 10.1016/j.semcancer.2023.06.001 PMC10526958

[4] Glennon KI Endo M Usui Y. Germline susceptibility to renal cell carcinoma and implications for genetic screening. JCO Precis Oncol 2024;8:e2400094. 39088769 10.1200/PO.24.00094

[5] Rinaldi L Senatore E Feliciello S. Kidney cancer: from tumor biology to innovative therapeutics. Biochim Biophys Acta Rev Cancer 2025;1880:189240. 39674419 10.1016/j.bbcan.2024.189240

[6] Zhang Q Ren H Ge L. A review on the role of long non-coding RNA and microRNA network in clear cell renal cell carcinoma and its tumor microenvironment. Cancer Cell Int 2023;23:16. 36732762 10.1186/s12935-023-02861-6 PMC9893571

[7] Rathmell WK Rumble RB Van Veldhuizen PJ. Management of metastatic clear cell renal cell carcinoma: ASCO Guideline. J Clin Oncol 2022;40:2957–95. 35728020 10.1200/JCO.22.00868

[8] Faes S Demartines N Dormond O. Mechanistic target of rapamycin inhibitors in renal cell carcinoma: potential, limitations, and perspectives. Front Cell Dev Biol 2021;9:636037. 33791295 10.3389/fcell.2021.636037 PMC8005589

[9] Jin J Xie Y Zhang JS. Sunitinib resistance in renal cell carcinoma: from molecular mechanisms to predictive biomarkers. Drug Resist Updat 2023;67:100929. 36739809 10.1016/j.drup.2023.100929

[10] Sato Y Yoshizato T Shiraishi Y. Integrated molecular analysis of clear-cell renal cell carcinoma. Nat Genet 2013;45:860–67. 23797736 10.1038/ng.2699

[11] Li Q Zhang Z Fan Y. Epigenetic alterations in renal cell cancer with TKIs resistance: from mechanisms to clinical applications. Front Genet 2020;11:562868. 33510766 10.3389/fgene.2020.562868 PMC7835797

[12] Wang Y Liu X Gong L. Mechanisms of sunitinib resistance in renal cell carcinoma and associated opportunities for therapeutics. Br J Pharmacol 2023;180:2937–55. 37740648 10.1111/bph.16252

[13] Bi K He MX Bakouny Z. Tumor and immune reprogramming during immunotherapy in advanced renal cell carcinoma. Cancer Cell 2021;39:649–661e5. 33711272 10.1016/j.ccell.2021.02.015 PMC8115394

[14] Lu Z Pan Y Wang S. Multi-omics and immunogenomics analysis revealed PFKFB3 as a targetable hallmark and mediates sunitinib resistance in papillary renal cell carcinoma: in silico study with laboratory verification. Eur J Med Res 2024;29:236. 38622715 10.1186/s40001-024-01808-5 PMC11017615

[15] Di SC Chen WJ Yang W. DEPDC1 as a metabolic target regulates glycolysis in renal cell carcinoma through AKT/mTOR/HIF1alpha pathway. Cell Death Dis 2024;15:533. 39068164 10.1038/s41419-024-06913-1 PMC11283501

[16] Zhang J Peng Q Fan J. Single-cell and spatial transcriptomics reveal SPP1-CD44 signaling drives primary resistance to immune checkpoint inhibitors in RCC. J Transl Med 2024;22:1157. 39736762 10.1186/s12967-024-06018-5 PMC11687132

[17] Agha RA Mathew G Rashid R. Transparency in the reporting of Artificial Intelligence – the TITAN guideline. Prem J Sci 2025;10:100082.

[18] Rose TL Kim WY. Renal cell carcinoma: a review. Jama 2024;332:1001–10. 39196544 10.1001/jama.2024.12848 PMC11790279

[19] Jiang A Li J He Z. Renal cancer: signaling pathways and advances in targeted therapies. MedComm (2020) 2024;5:e676. 39092291 10.1002/mco2.676 PMC11292401

[20] Xie D Li G Zheng Z. The molecular code of kidney cancer: a path of discovery for gene mutation and precision therapy. Mol Aspects Med 2025;101:101335. 39746268 10.1016/j.mam.2024.101335

[21] Hsieh JJ Chen D Wang PI. Genomic biomarkers of a randomized trial comparing first-line everolimus and sunitinib in patients with metastatic renal cell carcinoma. Eur Urol 2017;71:405–14. 27751729 10.1016/j.eururo.2016.10.007 PMC5431298

[22] Kwiatkowski DJ Choueiri TK Fay AP. Mutations in TSC1, TSC2, and MTOR are associated with response to rapalogs in patients with metastatic renal cell carcinoma. Clin Cancer Res 2016;22:2445–52. 26831717 10.1158/1078-0432.CCR-15-2631 PMC4976069

[23] Cancer Genome Atlas Research N. Comprehensive molecular characterization of clear cell renal cell carcinoma. Nature 2013;499:43–49. 23792563 10.1038/nature12222 PMC3771322

[24] Fenner A. Genetics: a molecular atlas of clear cell renal cell carcinoma. Nat Rev Clin Oncol 2013;10:485. 23836316 10.1038/nrclinonc.2013.122

[25] Roldan-Romero JM Beuselinck B Santos M. PTEN expression and mutations in TSC1, TSC2 and MTOR are associated with response to rapalogs in patients with renal cell carcinoma. Int J Cancer 2020;146:1435–44. 31335987 10.1002/ijc.32579

[26] Liu XL Zhang GM Huang SS. PTEN loss confers sensitivity to rapalogs in clear cell renal cell carcinoma. Acta Pharmacol Sin 2022;43:2397–409. 35165399 10.1038/s41401-022-00862-1 PMC9433447

[27] Voss MH Chen D Reising A. PTEN expression, not mutation status in TSC1, TSC2, or mTOR, correlates with the outcome on everolimus in patients with renal cell carcinoma treated on the randomized RECORD-3 trial. Clin Cancer Res 2019;25:506–14. 30327302 10.1158/1078-0432.CCR-18-1833

[28] Motzer RJ Barrios CH Kim TM. Phase II randomized trial comparing sequential first-line everolimus and second-line sunitinib versus first-line sunitinib and second-line everolimus in patients with metastatic renal cell carcinoma. J Clin Oncol 2014;32:2765–72. 25049330 10.1200/JCO.2013.54.6911 PMC5569681

[29] Eisele JW Frisino J Haglund W. Teenage suicide in King County, Washington. II. Comparison with adult suicides. Am J Forensic Med Pathol 1987;8:210–16. 3673980 10.1097/00000433-198708030-00003

[30] Sharma R Kadife E Myers M. Determinants of resistance to VEGF-TKI and immune checkpoint inhibitors in metastatic renal cell carcinoma. J Exp Clin Cancer Res 2021;40:186. 34099013 10.1186/s13046-021-01961-3 PMC8183071

[31] Sun F Chen Z Yao P. Meta-analysis of ABCG2 and ABCB1 polymorphisms with sunitinib-induced toxicity and efficacy in renal cell carcinoma. Front Pharmacol 2021;12:641075. 33762959 10.3389/fphar.2021.641075 PMC7982400

[32] Diekstra MH Swen JJ Boven E. CYP3A5 and ABCB1 polymorphisms as predictors for sunitinib outcome in metastatic renal cell carcinoma. Eur Urol 2015;68:621–29. 25930089 10.1016/j.eururo.2015.04.018

[33] van der Veldt AA Eechoute K Gelderblom H. Genetic polymorphisms associated with a prolonged progression-free survival in patients with metastatic renal cell cancer treated with sunitinib. Clin Cancer Res 2011;17:620–29. 21097692 10.1158/1078-0432.CCR-10-1828

[34] Cebrian A Gomez del Pulgar T Méndez-Vidal MJ. Functional PTGS2 polymorphism-based models as novel predictive markers in metastatic renal cell carcinoma patients receiving first-line sunitinib. Sci Rep 2017;7:41371. 28117391 10.1038/srep41371 PMC5259767

[35] Beuselinck B Jean-Baptiste J Schöffski P. Validation of VEGFR1 rs9582036 as predictive biomarker in metastatic clear-cell renal cell carcinoma patients treated with sunitinib. BJU Int 2016;118:890–901. 27417418 10.1111/bju.13585

[36] Beuselinck B Karadimou A Lambrechts D. Single-nucleotide polymorphisms associated with outcome in metastatic renal cell carcinoma treated with sunitinib. Br J Cancer 2013;108:887–900. 23462807 10.1038/bjc.2012.548 PMC3590652

[37] Garcia-Donas J Esteban E Leandro-García LJ. Single nucleotide polymorphism associations with response and toxic effects in patients with advanced renal-cell carcinoma treated with first-line sunitinib: a multicentre, observational, prospective study. Lancet Oncol 2011;12:1143–50. 22015057 10.1016/S1470-2045(11)70266-2

[38] Liu X Swen JJ Diekstra MH. A genetic polymorphism in CTLA-4 is associated with overall survival in sunitinib-treated patients with clear cell metastatic renal cell carcinoma. Clin Cancer Res 2018;24:2350–56. 29490989 10.1158/1078-0432.CCR-17-2815

[39] Diekstra MHM Swen JJ van der Zanden LF. Genome-wide meta-analysis identifies variants in DSCAM and PDLIM3 that correlate with efficacy outcomes in metastatic renal cell carcinoma patients treated with sunitinib. Cancers (Basel) 2022;14:2838. 35740506 10.3390/cancers14122838 PMC9220885

[40] Wolf MM Madden MZ Arner EN. VHL loss reprograms the immune landscape to promote an inflammatory myeloid microenvironment in renal tumorigenesis. J Clin Invest 2024;134:e173934. 10.1172/JCI173934 PMC11014672 38618956

[41] Labrousse-Arias D Martínez-Alonso E Corral-Escariz M. VHL promotes immune response against renal cell carcinoma via NF-kappaB-dependent regulation of VCAM-1. J Cell Biol 2017;216:835–47. 28235946 10.1083/jcb.201608024 PMC5350518

[42] Jiao M Hu M Pan D. VHL loss enhances antitumor immunity by activating the anti-viral DNA-sensing pathway. iScience 2024;27:110285. 39050705 10.1016/j.isci.2024.110285 PMC11267025

[43] Xu Z Liu L Jiang W. VHL missense mutation delineate aggressive clear cell renal cell carcinoma subtype with favorable immunotherapeutic response. J Immunother Cancer 2024;12:e009963. 39448203 10.1136/jitc-2024-009963 PMC11499804

[44] Liu XD Kong W Peterson CB. PBRM1 loss defines a nonimmunogenic tumor phenotype associated with checkpoint inhibitor resistance in renal carcinoma. Nat Commun 2020;11:2135. 32358509 10.1038/s41467-020-15959-6 PMC7195420

[45] Liu T Kong W Peterson CB. CCL5-dependent mast cell infiltration into the tumor microenvironment in clear cell renal cell carcinoma patients. Aging (Albany NY 2020;12:21809–36. 33177244 10.18632/aging.103999 PMC7695370

[46] Motzer RJ Banchereau R Hamidi H. Molecular subsets in renal cancer determine outcome to checkpoint and angiogenesis blockade. Cancer Cell 2020;38:803–817e4. 33157048 10.1016/j.ccell.2020.10.011 PMC8436590

[47] Sellner F Thalhammer S Klimpfinger M. Isolated pancreatic metastases of renal cell carcinoma-clinical particularities and seed and soil hypothesis. Cancers (Basel) 2023;15:339. 36672289 10.3390/cancers15020339 PMC9857376

[48] Bakouny Z Sadagopan A Ravi P. Integrative clinical and molecular characterization of translocation renal cell carcinoma. Cell Rep 2022;38:110190. 34986355 10.1016/j.celrep.2021.110190 PMC9127595

[49] Dawson MA Kouzarides T. Cancer epigenetics: from mechanism to therapy. Cell 2012;150:12–27. 22770212 10.1016/j.cell.2012.06.013

[50] Recillas-Targa F. Cancer epigenetics: an overview. Arch Med Res 2022;53:732–40. 36411173 10.1016/j.arcmed.2022.11.003

[51] Mehdi A Riazalhosseini Y. Epigenome aberrations: emerging driving factors of the clear cell renal cell carcinoma. Int J Mol Sci 2017;18:1774. 28812986 10.3390/ijms18081774 PMC5578163

[52] Joosten SC Smits KM Aarts MJ. Epigenetics in renal cell cancer: mechanisms and clinical applications. Nat Rev Urol 2018;15:430–51. 29867106 10.1038/s41585-018-0023-z

[53] Sekino Y Teishima J Liang G. Molecular mechanisms of resistance to tyrosine kinase inhibitor in clear cell renal cell carcinoma. Int J Urol 2022;29:1419–28. 36122306 10.1111/iju.15042 PMC10087189

[54] Shenoy N Vallumsetla N Zou Y. Role of DNA methylation in renal cell carcinoma. J Hematol Oncol 2015;8:88. 26198328 10.1186/s13045-015-0180-y PMC4511443

[55] Herman JG Latif F Weng Y. Silencing of the VHL tumor-suppressor gene by DNA methylation in renal carcinoma. Proc Natl Acad Sci U S A 1994;91:9700–04. 7937876 10.1073/pnas.91.21.9700 PMC44884

[56] Gossage L Eisen T. Alterations in VHL as potential biomarkers in renal-cell carcinoma. Nat Rev Clin Oncol 2010;7:277–88. 20368728 10.1038/nrclinonc.2010.42

[57] Krieg M Haas R Brauch H. Up-regulation of hypoxia-inducible factors HIF-1alpha and HIF-2alpha under normoxic conditions in renal carcinoma cells by von Hippel-Lindau tumor suppressor gene loss of function. Oncogene 2000;19:5435–43. 11114720 10.1038/sj.onc.1203938

[58] Stewart GD Powles T Van Neste C. Dynamic epigenetic changes to VHL occur with sunitinib in metastatic clear cell renal cancer. Oncotarget 2016;7:25241–50. 27029034 10.18632/oncotarget.8308 PMC5041900

[59] Stewart GD O'Mahony FC Laird A. Sunitinib Treatment exacerbates intratumoral heterogeneity in metastatic renal cancer. Clin Cancer Res 2015;21:4212–23. 26015515 10.1158/1078-0432.CCR-15-0207

[60] Choueiri TK Fay AP Gagnon R. The role of aberrant VHL/HIF pathway elements in predicting clinical outcome to pazopanib therapy in patients with metastatic clear-cell renal cell carcinoma. Clin Cancer Res 2013;19:5218–26. 23881929 10.1158/1078-0432.CCR-13-0491 PMC4522695

[61] Zhao T Bao Y Gan X. DNA methylation-regulated QPCT promotes sunitinib resistance by increasing HRAS stability in renal cell carcinoma. Theranostics 2019;9:6175–90. 31534544 10.7150/thno.35572 PMC6735520

[62] Zhao T Zhou Y Wang Q. QPCT regulation by CTCF leads to sunitinib resistance in renal cell carcinoma by promoting angiogenesis. Int J Oncol 2021;59:48. 34036385 10.3892/ijo.2021.5228 PMC8208629

[63] Kim JY Hwang J Lee SH. Decreased efficacy of drugs targeting the vascular endothelial growth factor pathway by the epigenetic silencing of FLT1 in renal cancer cells. Clin Clin Epigenet 2015;7:99. 10.1186/s13148-015-0134-9 PMC4572656 26380584

[64] Xiong Z Yuan C Xiong W. Restoring the epigenetically silenced PCK2 suppresses renal cell carcinoma progression and increases sensitivity to sunitinib by promoting endoplasmic reticulum stress. Theranostics 2020;10:11444–61. 33052225 10.7150/thno.48469 PMC7546001

[65] Li X Yu Q. PON1 hypermethylation is associated with progression of renal cell carcinoma. J Cell Mol Med 2019;23:6646–57. 31400051 10.1111/jcmm.14537 PMC6787518

[66] Dubrowinskaja N Gebauer K Peters I. Neurofilament heavy polypeptide CpG island methylation associates with prognosis of renal cell carcinoma and prediction of antivascular endothelial growth factor therapy response. Cancer Med 2014;3:300–09. 24464810 10.1002/cam4.181 PMC3987080

[67] Peters I Dubrowinskaja N Abbas M. DNA methylation biomarkers predict progression-free and overall survival of metastatic renal cell cancer (mRCC) treated with antiangiogenic therapies. PLoS One 2014;9:e91440. 24633192 10.1371/journal.pone.0091440 PMC3954691

[68] Pompas-Veganzones N Sandonis V Perez-Lanzac A. Myopodin methylation is a prognostic biomarker and predicts antiangiogenic response in advanced kidney cancer. Tumour Biol 2016;37:14301–10. 27592258 10.1007/s13277-016-5267-8

[69] Wang X Zhu W Long Q. The prognostic value and immune correlation of IL18 expression and promoter methylation in renal cell carcinoma. Clin Clin Epigenet 2023;15:14. 10.1186/s13148-023-01426-8 PMC9883904 36707882

[70] Lu X Vano Y Helleux A. An enhancer demethylator phenotype converged to immune dysfunction and resistance to immune checkpoint inhibitors in clear-cell renal cell carcinomas. Clin Cancer Res 2023;29:1279–91. 36374555 10.1158/1078-0432.CCR-22-2133

[71] Bai D Cheng Y Lu X. DNA methylation modification patterns identify distinct prognosis and responses to immunotherapy and targeted therapy in renal cell carcinoma. Front Biosci (Landmark Ed) 2023;28:224. 37796712 10.31083/j.fbl2809224

[72] de Cubas AA Dunker W Zaninovich A. DNA hypomethylation promotes transposable element expression and activation of immune signaling in renal cell cancer. JCI Insight 2020;5:e137569. 32493845 10.1172/jci.insight.137569 PMC7308050

[73] Wang J Zhang W Hou W. Molecular characterization, tumor microenvironment association, and drug susceptibility of DNA methylation-driven genes in renal cell carcinoma. Front Cell Dev Biol 2022;10:837919. 35386197 10.3389/fcell.2022.837919 PMC8978676

[74] Klumper N Ralser DJ Zarbl R. CTLA4 promoter hypomethylation is a negative prognostic biomarker at initial diagnosis but predicts response and favorable outcome to anti-PD-1 based immunotherapy in clear cell renal cell carcinoma. J Immunother Cancer 2021;9:e002949. 34446578 10.1136/jitc-2021-002949 PMC8395367

[75] Li L Zeng X Chao Z. Targeting alpha-ketoglutarate disruption overcomes immunoevasion and improves PD-1 blockade immunotherapy in renal cell carcinoma. Adv Sci (Weinh) 2023;10:e2301975. 37526345 10.1002/advs.202301975 PMC10520657

[76] Grimaldi AM Affinito O Salvatore M. CBX family members in two major subtypes of renal cell carcinoma: a comparative bioinformatic analysis. Diagnostics (Basel) 2022;12. 10.3390/diagnostics12102452 PMC9600067 36292141

[77] Jones N. Structure and function of transcription factors. Semin Cancer Biol 1990;1:5–17. 2133111

[78] Kamieniarz-Gdula K Proudfoot NJ. Transcriptional control by premature termination: a forgotten mechanism. Trends Genet 2019;35:553–64. 31213387 10.1016/j.tig.2019.05.005 PMC7471841

[79] Lambert SA Jolma A Campitelli LF. The human transcription factors. Cell 2018;172:650–65. 29425488 10.1016/j.cell.2018.01.029 PMC12908702

[80] Mattei AL Bailly N Meissner A. DNA methylation: a historical perspective. Trends Genet 2022;38:676–707. 35504755 10.1016/j.tig.2022.03.010

[81] de Klerk E Hoen PAT. Alternative mRNA transcription, processing, and translation: insights from RNA sequencing. Trends Genet 2015;31:128–39. 25648499 10.1016/j.tig.2015.01.001

[82] Tarau D Grünberger F Pilsl M. Structural basis of archaeal RNA polymerase transcription elongation and Spt4/5 recruitment. Nucleic Acids Res 2024;52:6017–35. 38709902 10.1093/nar/gkae282 PMC11162788

[83] Luan J Vermunt MW Syrett CM. CTCF blocks antisense transcription initiation at divergent promoters. Nat Struct Mol Biol 2022;29:1136–44. 36369346 10.1038/s41594-022-00855-y PMC10015438

[84] Xie Y Zhang Y Han J. The intronic cis element se1 recruits trans-acting repressor complexes to repress the expression of ELONGATED UPPERMOST INTERNODE1 in rice. Mol Plant 2018;11:720–35. 29524649 10.1016/j.molp.2018.03.001

[85] Hubner MR Spector DL. Chromatin dynamics. Annu Rev Biophys 2010;39:471–89. 20462379 10.1146/annurev.biophys.093008.131348 PMC2894465

[86] Su H Yang F Fu R. Cancer cells escape autophagy inhibition via NRF2-induced macropinocytosis. Cancer Cell 2021;39:678–693e11. 33740421 10.1016/j.ccell.2021.02.016 PMC8119368

[87] Weiss-Sadan T Ge M Hayashi M. NRF2 activation induces NADH-reductive stress, providing a metabolic vulnerability in lung cancer. Cell Metab 2023;35:487–503e7. 36841242 10.1016/j.cmet.2023.01.012 PMC9998367

[88] Chang K Chen Y Zhang X. DPP9 stabilizes NRF2 to suppress ferroptosis and induce sorafenib resistance in clear cell renal cell carcinoma. Cancer Res 2023;83:3940–55. 37713596 10.1158/0008-5472.CAN-22-4001

[89] Wang Q Gao S Shou Y. AIM2 promotes renal cell carcinoma progression and sunitinib resistance through FOXO3a-ACSL4 axis-regulated ferroptosis. Int J Biol Sci 2023;19:1266–83. 36923928 10.7150/ijbs.79853 PMC10008700

[90] He W Cheng F Zheng B. NUPR1 is a novel potential biomarker and confers resistance to sorafenib in clear cell renal cell carcinoma by increasing stemness and targeting the PTEN/AKT/mTOR pathway. Aging (Albany NY) 2021;13:14015–38. 34030133 10.18632/aging.203012 PMC8202846

[91] Jiang Z Yang G Wang G. SEC14L3 knockdown inhibited clear cell renal cell carcinoma proliferation, metastasis and sunitinib resistance through an SEC14L3/RPS3/NFkappaB positive feedback loop. J Exp Clin Cancer Res 2024;43:288. 39425205 10.1186/s13046-024-03206-5 PMC11490128

[92] Li W Ye K Li X. YTHDC1 is downregulated by the YY1/HDAC2 complex and controls the sensitivity of ccRCC to sunitinib by targeting the ANXA1-MAPK pathway. J Exp Clin Cancer Res 2022;41:250. 35974388 10.1186/s13046-022-02460-9 PMC9382764

[93] Xie J Yang Y Gao Y. Cuproptosis: mechanisms and links with cancers. Mol Cancer 2023;22:46. 36882769 10.1186/s12943-023-01732-y PMC9990368

[94] Chen L Min J Wang F. Copper homeostasis and cuproptosis in health and disease. Signal Transduct Target Ther 2022;7:378. 36414625 10.1038/s41392-022-01229-y PMC9681860

[95] Wang X Jia JH Zhang M. Adrenomedullin/FOXO3 enhances sunitinib resistance in clear cell renal cell carcinoma by inhibiting FDX1 expression and cuproptosis. FASEB J 2023;37:e23143. 37698353 10.1096/fj.202300474R

[96] Ruan H Li S Bao L. Enhanced YB1/EphA2 axis signaling promotes acquired resistance to sunitinib and metastatic potential in renal cell carcinoma. Oncogene 2020;39:6113–28. 32814829 10.1038/s41388-020-01409-6 PMC7498371

[97] Shou Y Yue C Wang Q. circPTPN12 promotes the progression and sunitinib resistance of renal cancer via hnRNPM/IL-6/STAT3 pathway. Cell Death Dis 2023;14:232. 37002206 10.1038/s41419-023-05717-z PMC10066201

[98] Huang S Hu J Hu M. Cooperation between SIX1 and DHX9 transcriptionally regulates integrin-focal adhesion signaling mediated metastasis and sunitinib resistance in KIRC. Oncogene 2024;43:2951–69. 39174859 10.1038/s41388-024-03126-w

[99] He H Li J Wang W. The SIRT7-mediated deacetylation of CHD1L amplifies HIF-2alpha-dependent signal that drives renal cell carcinoma progression and sunitinib resistance. Cell Biosci 2023;13:166. 37691108 10.1186/s13578-023-01113-4 PMC10493023

[100] He M Yang H Shi H. Sunitinib increases the cancer stem cells and vasculogenic mimicry formation via modulating the lncRNA-ECVSR/ERbeta/Hif2-alpha signaling. Cancer Lett 2022;524:15–28. 34461182 10.1016/j.canlet.2021.08.028

[101] Gu J Zhang Y Han Z. Targeting the ERbeta/Angiopoietin-2/Tie-2 signaling-mediated angiogenesis with the FDA-approved anti-estrogen faslodex to increase the sunitinib sensitivity in RCC. Cell Death Dis 2020;11:367. 32409702 10.1038/s41419-020-2486-0 PMC7224303

[102] Wang Y Peng M Zhong Y. The E3 ligase RBCK1 reduces the sensitivity of ccRCC to sunitinib through the ANKRD35-MITD1-ANXA1 axis. Oncogene 2023;42:952–66. 36732658 10.1038/s41388-023-02613-w

[103] Zhu L Ding R Yan H. ZHX2 drives cell growth and migration via activating MEK/ERK signal and induces sunitinib resistance by regulating the autophagy in clear cell renal cell carcinoma. Cell Death Dis 2020;11:337. 32382017 10.1038/s41419-020-2541-x PMC7206010

[104] Guo X Li R Bai Q. TFE3-PD-L1 axis is pivotal for sunitinib resistance in clear cell renal cell carcinoma. J Cell Mol Med 2020;24:14441–52. 33145941 10.1111/jcmm.16066 PMC7753981

[105] Yao D Xia S Jin C. Feedback activation of GATA1/miR-885-5p/PLIN3 pathway decreases sunitinib sensitivity in clear cell renal cell carcinoma. Cell Cycle 2020;19:2195–206. 32783497 10.1080/15384101.2020.1801189 PMC7513838

[106] Liu Y Cheng G Huang Z. Long noncoding RNA SNHG12 promotes tumour progression and sunitinib resistance by upregulating CDCA3 in renal cell carcinoma. Cell Death Dis 2020;11:515. 32641718 10.1038/s41419-020-2713-8 PMC7343829

[107] Shi H Sun Y He M. Targeting the TR4 nuclear receptor-mediated lncTASR/AXL signaling with tretinoin increases the sunitinib sensitivity to better suppress the RCC progression. Oncogene 2020;39:530–45. 31501521 10.1038/s41388-019-0962-8 PMC6962095

[108] Sun M Lughezzani G Perrotte P. Treatment of metastatic renal cell carcinoma. Nat Rev Urol 2010;7:327–38. 20458330 10.1038/nrurol.2010.57

[109] Cheng J Liu H Shen Y. Deubiquitinase UCHL1 stabilizes KDM4B to augment VEGF signaling and confer bevacizumab resistance in clear cell renal cell carcinoma. Transl Oncol 2024;45:101987. 38743986 10.1016/j.tranon.2024.101987 PMC11109002

[110] Schiavoni V Emanuelli M Campagna R. Paraoxonase-2 shRNA-mediated gene silencing suppresses proliferation and migration, while promotes chemosensitivity in clear cell renal cell carcinoma cell lines. J Cell Biochem 2024;125:e30572. 38706121 10.1002/jcb.30572

[111] Lu D Li Y Niu X. STAT2/SLC27A3/PINK1-mediated mitophagy remodeling lipid metabolism contributes to pazopanib resistance in clear cell renal cell carcinoma. Research (Wash D C) 2024;7:0539. 39600540 10.34133/research.0539 PMC11588985

[112] Xiao C Zhang W Hua M. RNF7 inhibits apoptosis and sunitinib sensitivity and promotes glycolysis in renal cell carcinoma via the SOCS1/JAK/STAT3 feedback loop. Cell Mol Biol Lett 2022;27:36. 35562668 10.1186/s11658-022-00337-5 PMC9107170

[113] Xu B Zhang J Ye L. Chinese herbal compound SanHuang decoction reverses axitinib resistance in ccRCC through regulating immune cell infiltration by affecting ADAMTS18 expression. Am J Cancer Res 2023;13:2841–60. 37560000 PMC10408491

[114] Yu YP Cai LC Wang XY. BMP8A promotes survival and drug resistance via Nrf2/TRIM24 signaling pathway in clear cell renal cell carcinoma. Cancer Sci 2020;111:1555–66. 32128917 10.1111/cas.14376 PMC7226287

[115] Cowman SJ Fuja DG Liu XD. Macrophage HIF-1alpha is an independent prognostic indicator in kidney cancer. Clin Cancer Res 2020;26:4970–82. 32586940 10.1158/1078-0432.CCR-19-3890 PMC7968518

[116] Sun J Tang Q Gao Y. VHL mutation-mediated SALL4 overexpression promotes tumorigenesis and vascularization of clear cell renal cell carcinoma via Akt/GSK-3beta signaling. J Exp Clin Cancer Res 2020;39:104. 32513235 10.1186/s13046-020-01609-8 PMC7278163

[117] Marona P Górka J Kwapisz O. Resistance to tyrosine kinase inhibitors promotes renal cancer progression through MCPIP1 tumor-suppressor downregulation and c-Met activation. Cell Death Dis 2022;13:814. 36138026 10.1038/s41419-022-05251-4 PMC9500022

[118] Wang J Zhao E Geng B. Downregulation of UBB potentiates SP1/VEGFA-dependent angiogenesis in clear cell renal cell carcinoma. Oncogene 2024;43:1386–96. 38467852 10.1038/s41388-024-03003-6 PMC11065696

[119] Dizman N Lyou Y Salgia N. Correlates of clinical benefit from immunotherapy and targeted therapy in metastatic renal cell carcinoma: comprehensive genomic and transcriptomic analysis. J Immunother Cancer 2020;8:e000953. 32661119 10.1136/jitc-2020-000953 PMC7359179

[120] Wang C Wang Y Hong T. Blocking the autocrine regulatory loop of Gankyrin/STAT3/CCL24/CCR3 impairs the progression and pazopanib resistance of clear cell renal cell carcinoma. Cell Death Dis 2020;11:117. 32051393 10.1038/s41419-020-2306-6 PMC7015941

[121] Hu X Chen L Liu T. TAF1D promotes tumorigenesis and metastasis by activating PI3K/AKT/mTOR signaling in clear cell renal cell carcinoma. Cell Signal 2024;124:111425. 39307376 10.1016/j.cellsig.2024.111425

[122] Choueiri TK Powles T Burotto M. Nivolumab plus cabozantinib versus sunitinib for advanced renal-cell carcinoma. N Engl J Med 2021;384:829–41. 33657295 10.1056/NEJMoa2026982 PMC8436591

[123] Rawat L Balan M Sasamoto Y. A novel combination therapy with cabozantinib and honokiol effectively inhibits c-Met-Nrf2-induced renal tumor growth through increased oxidative stress. Redox Biol 2023;68:102945. 37898101 10.1016/j.redox.2023.102945 PMC10628632

[124] Chen P Duan Y Lu X. RB1CC1 functions as a tumor-suppressing gene in renal cell carcinoma via suppression of PYK2 activity and disruption of TAZ-mediated PDL1 transcription activation. Cancer Immunol Immunother 2021;70:3261–75. 33837850 10.1007/s00262-021-02913-8 PMC10992581

[125] Zhang C Duan Y Xia M. TFEB mediates immune evasion and resistance to mTOR inhibition of renal cell carcinoma via induction of PD-L1. Clin Cancer Res 2019;25:6827–38. 31383732 10.1158/1078-0432.CCR-19-0733

[126] Wei S Krause HB Geynisman DM. Molecular characterization of TFE3-rearranged renal cell carcinoma: a comparative study with papillary and clear cell renal cell carcinomas. Mod Pathol 2024;37:100404. 38104891 10.1016/j.modpat.2023.100404

[127] Kong SK Kim BS Lim H. Dissection of PD-L1 promoter reveals differential transcriptional regulation of PD-L1 in VHL mutant clear cell renal cell carcinoma. Lab Invest 2022;102:352–62. 34789838 10.1038/s41374-021-00703-5

[128] Li J Sun H Fu M. TOPK mediates immune evasion of renal cell carcinoma via upregulating the expression of PD-L1. iScience 2023;26:107185. 37404377 10.1016/j.isci.2023.107185 PMC10316654

[129] Kim Y Yang H Lee WS. High levels of baseline serum IL-10 are associated with reduced clinical benefit from first-line immune checkpoint inhibitor therapy in advanced renal cell carcinoma. J Cancer 2023;14:935–42. 37151396 10.7150/jca.81384 PMC10158513

[130] Huseni MA Wang L Klementowicz JE. CD8(+) T cell-intrinsic IL-6 signaling promotes resistance to anti-PD-L1 immunotherapy. Cell Rep Med 2023;4:100878. 36599350 10.1016/j.xcrm.2022.100878 PMC9873827

[131] Hirsch L Flippot R Escudier B. Immunomodulatory roles of VEGF pathway inhibitors in renal cell carcinoma. Drugs 2020;80:1169–81. 32601914 10.1007/s40265-020-01327-7

[132] Wertheimer T Zwicky P Rindlisbacher L. IL-23 stabilizes an effector T(reg) cell program in the tumor microenvironment. Nat Immunol 2024;25:512–24. 38356059 10.1038/s41590-024-01755-7 PMC10907296

[133] Jarocki M Karska J Kowalski S. Interleukin 17 and its involvement in renal cell carcinoma. J Clin Med 2022;11:4973. 36078902 10.3390/jcm11174973 PMC9457171

[134] Uvarova AN Zheremyan EA Ustiugova AS. Autoimmunity-associated SNP rs3024505 disrupts STAT3 binding in B cells, leading to IL10 dysregulation. Int J Mol Sci 2024;25:10196. 39337678 10.3390/ijms251810196 PMC11432243

[135] Shi K Liu X Du G. In vivo antitumour activity of Britanin against gastric cancer through nuclear factor-kappaB-mediated immune response. J Pharm Pharmacol 2020;72:607–18. 31943207 10.1111/jphp.13230

[136] Courcol RJ Martin GR. In-vitro activity of the combination of ceftriaxone and fosfomycin against staphylococci. J Antimicrob Chemother 1987;19:276–78. 3571049 10.1093/jac/19.2.276

[137] Kim SJ Saeidi S Cho NC. Interaction of Nrf2 with dimeric STAT3 induces IL-23 expression: implications for breast cancer progression. Cancer Lett 2021;500:147–60. 33278500 10.1016/j.canlet.2020.11.047

[138] Huber M Brüstle A Reinhard K. IRF4 is essential for IL-21-mediated induction, amplification, and stabilization of the Th17 phenotype. Proc Natl Acad Sci U S A 2008;105:20846–51. 19088203 10.1073/pnas.0809077106 PMC2634912

[139] Santagata S Rea G Bello AM. Targeting CXCR4 impaired T regulatory function through PTEN in renal cancer patients. Br J Cancer 2024;130:2016–26. 38704478 10.1038/s41416-024-02702-x PMC11183124

[140] Pan J Mestas J Burdick MD. Stromal derived factor-1 (SDF-1/CXCL12) and CXCR4 in renal cell carcinoma metastasis. Mol Cancer 2006;5:56. 17083723 10.1186/1476-4598-5-56 PMC1636662

[141] Liu H Sun S Wang G. Tyrosine kinase inhibitor cabozantinib inhibits murine renal cancer by activating innate and adaptive immunity. Front Oncol 2021;11:663517. 33954115 10.3389/fonc.2021.663517 PMC8089383

[142] Wang B Jiang B Du L. Tumor-intrinsic RGS1 potentiates checkpoint blockade response via ATF3-IFNGR1 axis. Oncoimmunology 2023;12:2279800. 38264343 10.1080/2162402X.2023.2279800 PMC10804258

[143] Hu J Wang SG Hou Y. Multi-omic profiling of clear cell renal cell carcinoma identifies metabolic reprogramming associated with disease progression. Nat Genet 2024;56:442–57. 38361033 10.1038/s41588-024-01662-5 PMC10937392

[144] Chakraborty S Balan M Sabarwal A. Metabolic reprogramming in renal cancer: events of a metabolic disease. Biochim Biophys Acta Rev Cancer 2021;1876:188559. 33965513 10.1016/j.bbcan.2021.188559 PMC8349779

[145] Huang J Wang Y Xu F. Neoadjuvant toripalimab combined with axitinib in patients with locally advanced clear cell renal cell carcinoma: a single-arm, phase II trial. J Immunother Cancer 2024;12:e008475. 38862251 10.1136/jitc-2023-008475 PMC11168135

[146] Xiao J Wang S Chen L. 25-hydroxycholesterol regulates lysosome AMP kinase activation and metabolic reprogramming to educate immunosuppressive macrophages. Immunity 2024;57:1087–1104e7. 38640930 10.1016/j.immuni.2024.03.021

[147] Frumento G Rotondo R Tonetti M. Tryptophan-derived catabolites are responsible for inhibition of T and natural killer cell proliferation induced by indoleamine 2,3-dioxygenase. J Exp Med 2002;196:459–68. 12186838 10.1084/jem.20020121 PMC2196046

[148] Mellor AL Keskin DB Johnson T. Cells expressing indoleamine 2,3-dioxygenase inhibit T cell responses. J Immunol 2002;168: 3771–76. 11937528 10.4049/jimmunol.168.8.3771

[149] Holmgaard RB Zamarin D Li Y. Tumor-expressed IDO recruits and activates MDSCs in a treg-dependent manner. Cell Rep 2015;13:412–24. 26411680 10.1016/j.celrep.2015.08.077 PMC5013825

[150] Shu G Chen M Liao W. PABPC1L induces IDO1 to promote tryptophan metabolism and immune suppression in renal cell carcinoma. Cancer Res 2024;84:1659–79. 38382068 10.1158/0008-5472.CAN-23-2521 PMC11094425

[151] Diaz-Montero CM Rini BI Finke JH. The immunology of renal cell carcinoma. Nat Rev Nephrol 2020;16:721–35. 32733094 10.1038/s41581-020-0316-3

[152] Song X Zhu Y Geng W. Spatial and single-cell transcriptomics reveal cellular heterogeneity and a novel cancer-promoting treg cell subset in human clear-cell renal cell carcinoma. J Immunother Cancer 2025;13:e010183. 39755578 10.1136/jitc-2024-010183 PMC11748785

[153] van der Veeken J Campbell C Pritykin Y. Genetic tracing reveals transcription factor Foxp3-dependent and Foxp3-independent functionality of peripherally induced treg cells. Immunity 2022;55:1173–1184e7. 35700740 10.1016/j.immuni.2022.05.010 PMC9885886

[154] He M Zong X Xu B. Dynamic Foxp3-chromatin interaction controls tunable treg cell function. J Exp Med 2024;221:e20232068. 38935023 10.1084/jem.20232068 PMC11211070

[155] Xu S Hu X Chong Y. Investigating the role of FoxP3 in renal cell carcinoma metastasis with BAP1 or SEDT2 mutation. Int J Mol Sci 2023;24:12301. 37569676 10.3390/ijms241512301 PMC10419232

[156] Hirai H Yokota A Tamura A. Non-steady-state hematopoiesis regulated by the C/EBPbeta transcription factor. Cancer Sci 2015;106:797–802. 25940801 10.1111/cas.12690 PMC4520629

[157] Wang W Xia X Mao L. The CCAAT/enhancer-binding protein family: its roles in MDSC expansion and function. Front Immunol 2019;10:1804. 31417568 10.3389/fimmu.2019.01804 PMC6684943

[158] Bitsch R Kurzay A Kurt FÖ. STAT3 inhibitor napabucasin abrogates MDSC immunosuppressive capacity and prolongs survival of melanoma-bearing mice. J Immunother Cancer 2022;10:e004384. 35301236 10.1136/jitc-2021-004384 PMC8932276

[159] Sun Q Zhao X Li R. STAT3 regulates CD8+ T cell differentiation and functions in cancer and acute infection. J Exp Med 2023;220:e20220686. 36688918 10.1084/jem.20220686 PMC9884582

[160] Lee JM Hammarén HM Savitski MM. Control of protein stability by post-translational modifications. Nat Commun 2023;14:201. 36639369 10.1038/s41467-023-35795-8 PMC9839724

[161] Vu LD Gevaert K De Smet I. Protein language: post-translational modifications talking to each other. Trends Plant Sci 2018;23:1068–80. 30279071 10.1016/j.tplants.2018.09.004

[162] Mertins P Qiao JW Patel J. Integrated proteomic analysis of post-translational modifications by serial enrichment. Nat Methods 2013;10:634–37. 23749302 10.1038/nmeth.2518 PMC3943163

[163] Bridges KR Schmidt GJ Jensen MI. The acetylation of hemoglobin by aspirin. In vitro and in vivo. J Clin Invest 1975;56:201–07. 237937 10.1172/JCI108068 PMC436570

[164] Clarke SG. Protein methylation at the surface and buried deep: thinking outside the histone box. Trends Biochem Sci 2013;38:243–52. 23490039 10.1016/j.tibs.2013.02.004 PMC3634909

[165] Popovic D Vucic D Dikic I. Ubiquitination in disease pathogenesis and treatment. Nat Med 2014;20:1242–53. 25375928 10.1038/nm.3739

[166] Bilbrough T Piemontese E Seitz O. Dissecting the role of protein phosphorylation: a chemical biology toolbox. Chem Soc Rev 2022;51:5691–730. 35726784 10.1039/d1cs00991e

[167] Schjoldager KT Narimatsu Y Joshi HJ. Global view of human protein glycosylation pathways and functions. Nat Rev Mol Cell Biol 2020;21:729–49. 33087899 10.1038/s41580-020-00294-x

[168] SMesquita F Abrami L Linder ME. Mechanisms and functions of protein S-acylation. Nat Rev Mol Cell Biol 2024;25:488–509. 38355760 10.1038/s41580-024-00700-8 PMC12010433

[169] Shu F Xiao H Li QN. Epigenetic and post-translational modifications in autophagy: biological functions and therapeutic targets. Signal Transduct Target Ther 2023;8:32. 36646695 10.1038/s41392-022-01300-8 PMC9842768

[170] Deribe YL Pawson T Dikic I. Post-translational modifications in signal integration. Nat Struct Mol Biol 2010;17:666–72. 20495563 10.1038/nsmb.1842

[171] Sheng X Xia Z Yang H. The ubiquitin codes in cellular stress responses. Protein Cell 2024;15:157–90. 37470788 10.1093/procel/pwad045 PMC10903993

[172] Gavi F Sighinolfi MC Pallotta G. Multiomics in renal cell carcinoma: current landscape and future directions for precision medicine. Curr Urol Rep 2025;26:44. 40418294 10.1007/s11934-025-01276-2 PMC12106551

[173] Liao C Hu L Zhang Q. Von hippel-lindau protein signalling in clear cell renal cell carcinoma. Nat Rev Urol 2024;21:662–75. 38698165 10.1038/s41585-024-00876-w

[174] Prade JD De Souza RS D’αvila CM. An overview of renal cell carcinoma hallmarks, drug resistance, and adjuvant therapies. Cancer Diagn Progn 2023;3:616–34. 37927802 10.21873/cdp.10264 PMC10619564

[175] Liu Y Zhang H Fang Y. Non-coding RNAs in renal cell carcinoma: implications for drug resistance. Biomed Pharmacother 2023;164:115001. 37315433 10.1016/j.biopha.2023.115001

[176] Zhan Y Liu Y Yang R. CircPTEN suppresses human clear cell renal carcinoma progression and resistance to mTOR inhibitors by targeting epigenetic modification. Drug Resist Updat 2023;71:101003. 37866104 10.1016/j.drup.2023.101003

[177] Li M Li L Zheng J. Liquid biopsy at the frontier in renal cell carcinoma: recent analysis of techniques and clinical application. Mol Cancer 2023;22:37. 36810071 10.1186/s12943-023-01745-7 PMC9942319

[178] Chen G Zhang J Fu Q. Integrative analysis of multi-omics data for liquid biopsy. Br J Cancer 2023;128:505–18. 36357703 10.1038/s41416-022-02048-2 PMC9938261

[179] Chen L Huang L Gu Y. Lactate-lactylation hands between metabolic reprogramming and immunosuppression. Int J Mol Sci 2022;23:11943. 36233246 10.3390/ijms231911943 PMC9569569

[180] Yamaguchi H Hsu JM Yang WH. Mechanisms regulating PD-L1 expression in cancers and associated opportunities for novel small-molecule therapeutics. Nat Rev Clin Oncol 2022;19:287–305. 35132224 10.1038/s41571-022-00601-9

